# A Framework for Fast, Autonomous, and Reliable Tool Incorporation on iCub

**DOI:** 10.3389/frobt.2018.00098

**Published:** 2018-08-22

**Authors:** Tanis Mar, Vadim Tikhanoff, Lorenzo Natale

**Affiliations:** iCub Facility, Istituto Italiano di Tecnologia, Genoa, Italy

**Keywords:** tool use, code:cplusplus, tool incorporation, affordances, iCub, 3D reconstruction, humanoid, tool pose

## Abstract

One of the main advantages of building robots with size and motor capabilities close to those of humans, such as iCub, lies in the fact that they can potentially take advantage of a world populated with tools and devices designed by and for humans. However, in order to be able to do proper use of the tools around them, robots need to be able to *incorporate* these tools, that is, to build a representation of the tool's geometry, reach and pose with respect to the robot. The present paper tackles this argument by presenting a repository which implements a series of interconnected methods that enable autonomous, fast, and reliable tool incorporation on the iCub platform.

## 1. Overview

A critical problem in most studies of tool use in developmental robotics is that actions are performed without considering the geometry or pose of tools that the robot uses. Instead, most experiments apply standard grasps and assume pre-defined kinematic end-effector extensions that do not take into account the particular pose of the tool in the robot's hand (Gonçalves et al., 2014; Dehban et al., 2017). In order to overcome this limitation, this paper presents a repository which implements a series of interconnected methods that enable autonomous, fast, and reliable estimation of a tool's geometry, reach and pose with respect to the iCub's hand, in order to attach it to the robot's kinematic chain, thereby enabling dexterous tool use. Indeed, this methods have been successfully applied in the study presented in Mar et al. (2017).

The repository can be found at:

https://github.com/robotology/tool-incorporation

We name this process *tool incorporation* because of its meaning referring to embodiment (literally, *in-corpore*), as it enables iCub to build a representation of the tool with respect to, and included in, its own body representation. The iCub is a full body humanoid robot with 53 Degrees of Freedom (DoF) (Metta et al., 2010), including head, arms, and torso. The iCub software is structured as modules that communicate with each other using YARP middleware, which enables multi-machine and multi-platform integration (Metta, 2006). Modules provide specific functionalities, and work together in form of applications to achieve desired behaviors on the iCub. Vision is provided by the cameras mounted in the robot's eyes, from which stereo matching can be applied to estimate depth (Fanello et al., 2014). Image processing is achieved with the help of OpenCV and PCL libraries, for 2D and 3D processing respectively (Rusu and Cousins, 2011; Itseez, 2015). All the methods described in this paper are implemented as functions in the toolIncorporation module.

The remainder of this paper is structured according to the main methods required to incorporate tools. Section 2 describes the methods for tool recognition, or visual appearance learning if the tool has not been seen before. Section 3 presents a method that enables iCub to reconstruct a 3D representation of the tool in its hand using its stereo-vision capabilities. Section 4 explains the meaning and estimation of the tool's intrinsic frame and of the tooltip. Finally, section 5 details a method for faster estimation of a tool's pose when its model is available.

## 2. Tool recognition

The first step for tool incorporation is to recognize the tool in the robot's hand, so that its model can be loaded if the tool is known, or its visual appearance learned otherwise. To that end, the method applied in this work builds upon the techniques described in Pasquale et al. (2016). In that paper, a pre-trained CNN (AlexNet trained on imageNet, Krizhevsky et al., 2012) learned to associate a cropped image of an object presented by the experimenter with a provided label. In this work, we extended this approach in order to reduce the need of an external teacher, so that it is only required to hand over the tool to the robot and provide its label.

Once the iCub robot is grasping the tool in its hand, exploration is performed by moving it to different poses, so that it can be observed from different perspectives (implemented in function exploreTool). These poses are predefined to utilize the range of iCub's wrist joints to achieve distinct perspectives.

On each of the considered poses iCub focuses on the tool's effector, understood as the part of the tool that interacts with the environment. However, at this point the robot has no information about the tool's geometry or pose in order to estimate where the effector might be (these are discussed in section 4.3). Therefore, in order to locate the effector, iCub initially looks just slightly over its hand (10 cm along the X axis and –10 cm along the Y axis of the hand reference frame). Then, it locates the tooltip on the image by iteratively extracting the tool outline from the disparity map, and looking at the point in the blob further away from the hand reference frame. This process, which is implemented in function lookAtTool, is repeated until the position of the estimated tooltip is stable, or a given number of iterations has been surpassed.

Once iCub is correctly gazing at the tool effector, a series of images of the tool are obtained by cropping a region around the tool, which is determined by the bounding box of the closest blob obtained with dispBlobber^1^, plus a margin of 10 pixels on each side. Finally the cropped images are fed to a CNN whose output feeds in turn a linear classifier which associates them to the user provided tool label. This process is performed by the onTheFlyRecognition application, which is called from by the learn function provided with the tool label.

This sequence –tool effector location and subsequent cropping of the tool region to feed the CNN– is repeated for all the exploration poses considered, which provides enough perspectives to recognize the tool in any future pose in which it might be grasped in the future.

After the visual appearance of the set of available tools has been learned, the process of classification is simple. After iCub is given any tool, it observes it in any of the exploratory poses and uses the same method to crop it from the rest of the image. The cropped image is in turn sent to the trained classifier (in this case, using the recognize function), which returns the estimated label of the tool. It should be noted that tools can be learned in either terms of instances or categories. In the first case, the user should provide a distinct label for each individual tool given to iCub, and an associated pointcloud model. In the second case, tools of the same category (e.g., rakes, sticks, shovels), should be given the generic label of that category, and a generic model of the tool category provided.

## 3. Tool 3d reconstruction

In cases where a 3D model of the tool is not available, the robot should be able to reconstruct it through exploration. In this section we describe an approach that allows iCub to achieve this, without the need of external intervention by the experimenter. Essentially, it consists of iterative segmentation, reconstruction, and merge of the tool's partial views from different perspectives.

Similar techniques have been presented in many different papers in the recent years (Ren et al., 2013; Zhang et al., 2015). However, most of these studies assume either a fixed camera and an object being moved externally (by the user or on a turning table), which could not be considered autonomous; or a fix scene and a moving camera/robot navigating around it, which is unfeasible on the current iCub setup. Therefore, in the present work we implemented a method by means of which iCub can reconstruct a tool's complete pointcloud representation by obtaining partial view reconstructions from different perspectives and incrementally merging them together.

The method applied to observe the tool effector is analogous to the one described in the previous section for learning the tool's visual appearance, and in fact, both processes can be run simultaneously (by calling the exploreTool function with the 2D and 3D flags active). For reconstruction, the steps performed at each exploration pose are the following:

- Segmentation:
  After the gaze is properly oriented toward the tool effector, as described in section 2, instead of just cropping the bounding box around the tool, the tool blob is segmented with the dispBlobber module, which returns the pixels in the image that correspond to the tool.
- Reconstruction:
  This list of pixels is sent to the seg2cloud module, which computes the 3D coordinates of each point in the robot reference frame and returns them as a pointcloud. This pointcloud is transformed from the robot frame to the hand's reference frame using the robot's kinematics, which greatly facilitates subsequent merging, as the hand provides a coherent reference frame for all the partial reconstructions.
  Moreover, we can safely assume that the tool is connected with the hand, and it does not extend beyond certain boundaries. Therefore, in order to remove any points on the reconstructed pointcloud that might belong to the background, the pointcloud is truncated in all three axes of the hand reference frame, removing all the points outside the (0.0, 35)cm range in the *X* axis, (−30, 0.0)cm range in the *Y* axis, and (−15, 15)cm range in the *Z* axis. Additionally, as in many cases part of the hand might also be present in the reconstructed pointcloud, it is removed by filtering out all the points in the which are inside a radius of 8 cm from the origin of the hand reference frame. Finally, the pointcloud is smoothed by applying a statistical filter for outlier removal. The described pointcloud reconstruction, transformation and filtering are performed by the getPointCloud function.
- Merging:
  Although all the partial reconstructed pointclouds are represented in a coherent reference frame, they are not perfectly aligned due to errors in depth estimation and robot kinematics. Therefore, a further refinement step is performed using the Iterative Closest Point algorithm (ICP) (Besl and McKay, 1992). We assume that the required refinement is small and thus discard as unsuccessful those cases in which the resulting roto-translation is larger than a given threshold. Finally, in order to merge overlapping surfaces and reduce noise, the resulting pointcloud is downsampled uniformly using a voxelized grid.

As a result of this process, a complete pointcloud representation of the explored tool is obtained, which also reflects the pose with which the tool is being grasped by iCub. We refer to this representation as an **oriented pointcloud model**, that is, the available pointcloud model of the tool being held by the robot, whose coordinates match the position of the actual tool with respect to the robot's hand reference frame.

## 4. Tool reference frame and tooltip estimation

Although the pose of the oriented pointcloud model corresponds to that of the tool in the robot's hand, its orientation is not readily available for the robot, as it is only implicit in the pointcloud representation. In the present section we present a method to make this information explicit, based on the definition and estimation of a reference frame intrinsic to each tool, applicable to the vast majority of man-made tools that could be present in a robotic tool use scenario. This frame of reference, referred to as **tool intrinsic reference frame**, and denoted as **f**, identifies the effector and handle of the tool, provides its orientation with respect to the hand reference frame, and facilitates the computation of the tooltip's location.

### 4.1. Tool reference frame definition

**Figure 1:**
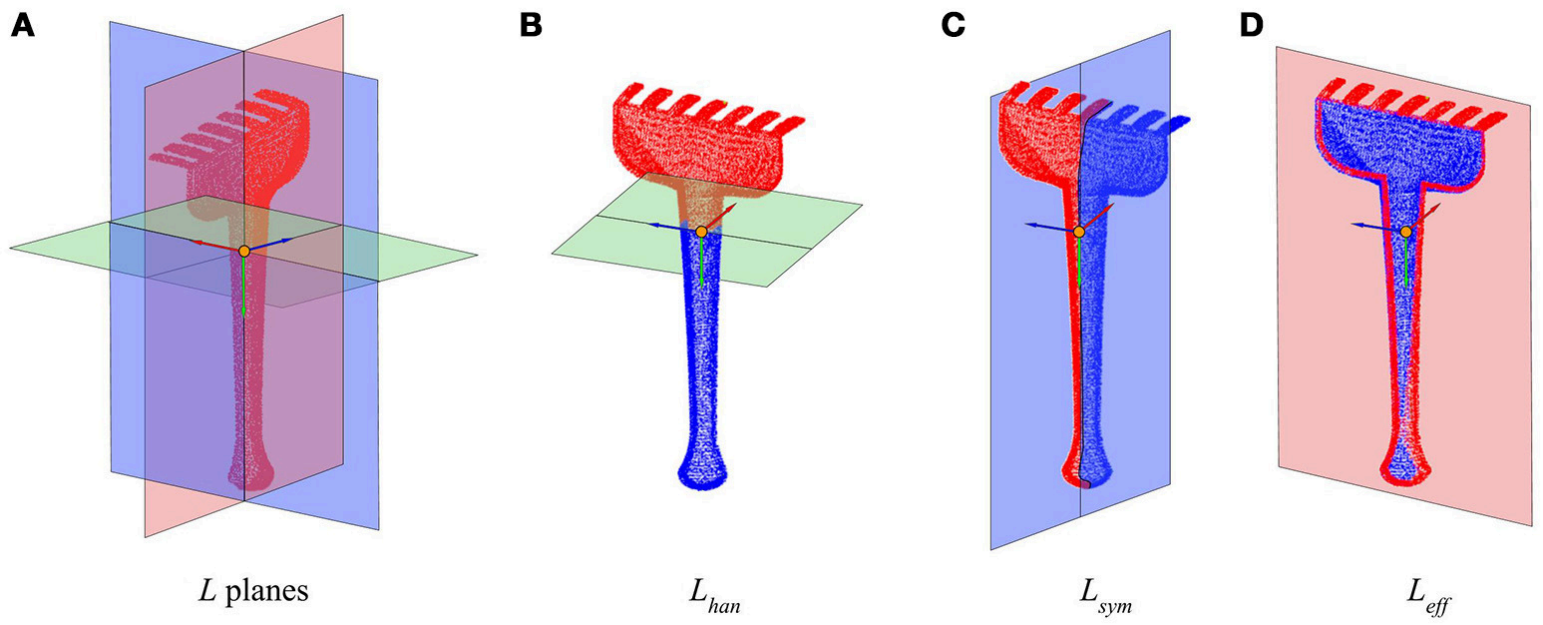
Planes and axes that determine the tool's intrinsic reference frame. **(A)** Tool model divided by its three characteristic *L* planes. **(B)** Handle plane *L*_*han*_. **(C)** Symmetry plane *L*_*sym*_. **(D)** Effector plane *L*_*eff*_. The reference frame in all figures shows **f**_*eff*_ in red, **f**_*han*_ in green, and **f**_*sym*_ in blue.

Given any radial tool^2^, generally we can define three orthogonal characteristic tool planes as can be observed in Figure 1, denoted together as a tool's *L* planes:

- **Handle plane** (*L*_*han*_): It is perpendicular to the handle axis, and divides the tool into the effector and the handle sides.
- **Symmetry plane** (*L*_*sym*_): It is the plane with respect to which the tool has the maximum symmetry. It runs along the handle and divides the tool into two equal (or almost) longitudinal halves.
- **Effector plane** (*L*_*eff*_): Orthogonal to the two previous planes, usually divides the “forward” and “back” sides of the tool, forward being the side where the effector is.

The planes' normal vectors can be chosen so that they define a right-hand reference frame, which we refer to as the **tool intrinsic reference frame**,(**f**). To this end, the origin and orientation of the corresponding axes is chosen so that they preserve the following characteristics:

- **Effector axis (***X***)** (**f**_*eff*_): It is positive in the direction of the effector, i.e., toward the “forward” side of the tool.
- **Handle axis (***Y***)** (**f**_*han*_): Is positive in the direction toward the handle, and negative in the direction toward the effector side of the tool.
- **Symmetry axis (***Z***)** (**f**_*sym*_): The symmetry basis vector is obtained as the outer product of the other two to ensure orthogonality, so it is positive on the “left” side of the tool, if the effector is looking “forward”.

### 4.2. Tool reference frame estimation

Based on the previous definitions, here we propose a method to automatically estimate the tool intrinsic reference frame **f** of a tool's pointcloud representation *W*, relying solely on the assumption that *W* represents an oriented pointcloud model, that is, it is expressed with respect to the hand reference frame of the robot. The proposed procedure consists on the following steps, which can be observed in function findSyms:

1. Find the pointcloud's main axes: The estimation of **f**'s origin and direction can be achieved by computing the covariance matrix of the pointcloud *W*. The origin is determined at the center of mass *o*, and the 3 eigenvectors **v** with larger eigenvalues λ correspond to the pointcloud's main axes:
  (1) $$\begin{array}{r} C=cov\left(W\right), \end{array}$$
  (2) $$\begin{array}{r} C\text{v}=\lambda \text{v}, \end{array}$$
  (3) $$\begin{array}{r} L\left[i\right]⊥\text{v}\left[i\right],i\in \left\{0,1,2\right\}. \end{array}$$
  Therefore, this set of orthogonal vectors **v** defines a set of orthogonal planes that approximate the tool planes *L*, but their correspondence with the specific planes defined above, as well as their orientation, need to be determined to fully characterize **f**.
2. Identify the planes:
  - Handle plane *L*_*han*_: The handle is situated along the longest tool dimension. Thus, the eigenvector with largest eigenvalue indicates the direction of the handle axis, normal to the Handle plane. That is,
    (4) $$\begin{array}{r} {\text{v}}_{han}=\text{v}\left[n\right],\text{ where}n=\underset{i\in \left\{0,1,2\right\}}{\arg\max}\left(\lambda \left[i\right]\right), \end{array}$$
    (5) $$\begin{array}{r} \text{accordingly,}L_{han}=L\left[n\right] \end{array}$$
  - Symmetry plane *L*_*sym*_: The symmetry plane corresponds by definition to the plane with respect to which the tool has the maximum symmetry. Thus:
    (6) $$L_{sym}=L[m],\text{ where }m=\;\underset{j\in \{0,1,2\}\neq n}{\text{arg max}}\;(sym(L[j]).$$
  - Effector plane *L*_*eff*_: The effector plane is computed in relation to previous two planes, as the plane orthogonal to both the Handle and the Symmetry plane:
    (7) $$\begin{array}{r} L_{eff}=L\left[k\right],\text{ where}k\in 0,1,2\neq n,m \end{array}$$
    (8) $$\begin{array}{r} L_{eff}⊥L_{sym}⊥L_{han} \end{array}$$
3. Find the axes orientations:
  - Handle axis **f**_*han*_: Determines the side where the handle of the tool is (opposite of the effector). Following the assumption that *W* is represented with respect to the hand reference frame, it follows that the handle is on the side of *L*_*han*_ that contains the origin of the pointcloud reference frame (i.e., the hand). Thus, the orientation of **f**_*han*_ is set so that the positive values correspond to the side of *L*_*han*_ that contains the origin.
  - Effector axis **f**_*eff*_: In order to determine the direction that corresponds with “forward” in a tool, we consider the saliency of the features on each side of the effector plane. Specifically, the “forward” side of the pointcloud *W* is defined as the side where the effector half of the tool (determined in the previous step) contains points further away from the tool's intrinsic reference frame origin *o*. Thus, the orientation of the effector axis **f**_*eff*_ (perpendicular to the effector plane) is set such that the positive values are located on the salient side of the effector plane.
  - Symmetry axis **f**_*sym*_: The orientation of **f**_*sym*_ is chosen so that the set of axes defined by **v** corresponds to a right-handed coordinate system. Thus, it is computed as the cross product between the handle and effector axes basis vectors:
    (9) $$\begin{array}{r} {\text{f}}_{sym}={\text{f}}_{han}\times {\text{f}}_{eff} \end{array}$$

The tool intrinsic reference frame **f** is actually expressed on the same frame of reference that the pointcloud reconstruction from which it is estimated, that is, the hand reference frame. Thus, the equations of the frame's axes represent explicitly the orientation of the tool in any of its three axis.

One of the strengths of this approach to estimate the tool's frame of reference **f** is that it relies on very few and general assumptions to be met in order to work successfully, namely, that the tool's handle axis is longer that any other axis, and that the tool has a certain degree of symmetry along a plane that contains that axis. Moreover, the method is also very robust to noise in the 3D representation of the tool, since all the computations required throughout the process of determining **f** have a high tolerance to noise. Indeed, as most of the decisions are made in terms of comparison (symmetry between two sides of a plane, longest axis, furthest away point), if noise affects the whole pointcloud similarly, it would not modify their outcome.

### 4.3. Tooltip estimation

As stated above, one of the main advantages of estimating the tool reference frame **f** is that it enables to precisely locate the tooltip, required to perform the extension of the robot's kinematic chain to the new end-effector provided by the tool. Thus, the tool tip is defined in terms of the concepts defined and estimated above:

**Tooltip:** Location on the tool represented by the point on the Symmetry plane of the tool, above the Handle plane (i.e., on the effector side), furthest away from the Effector plane, on the positive side of the effector axis.

**Figure 2:**
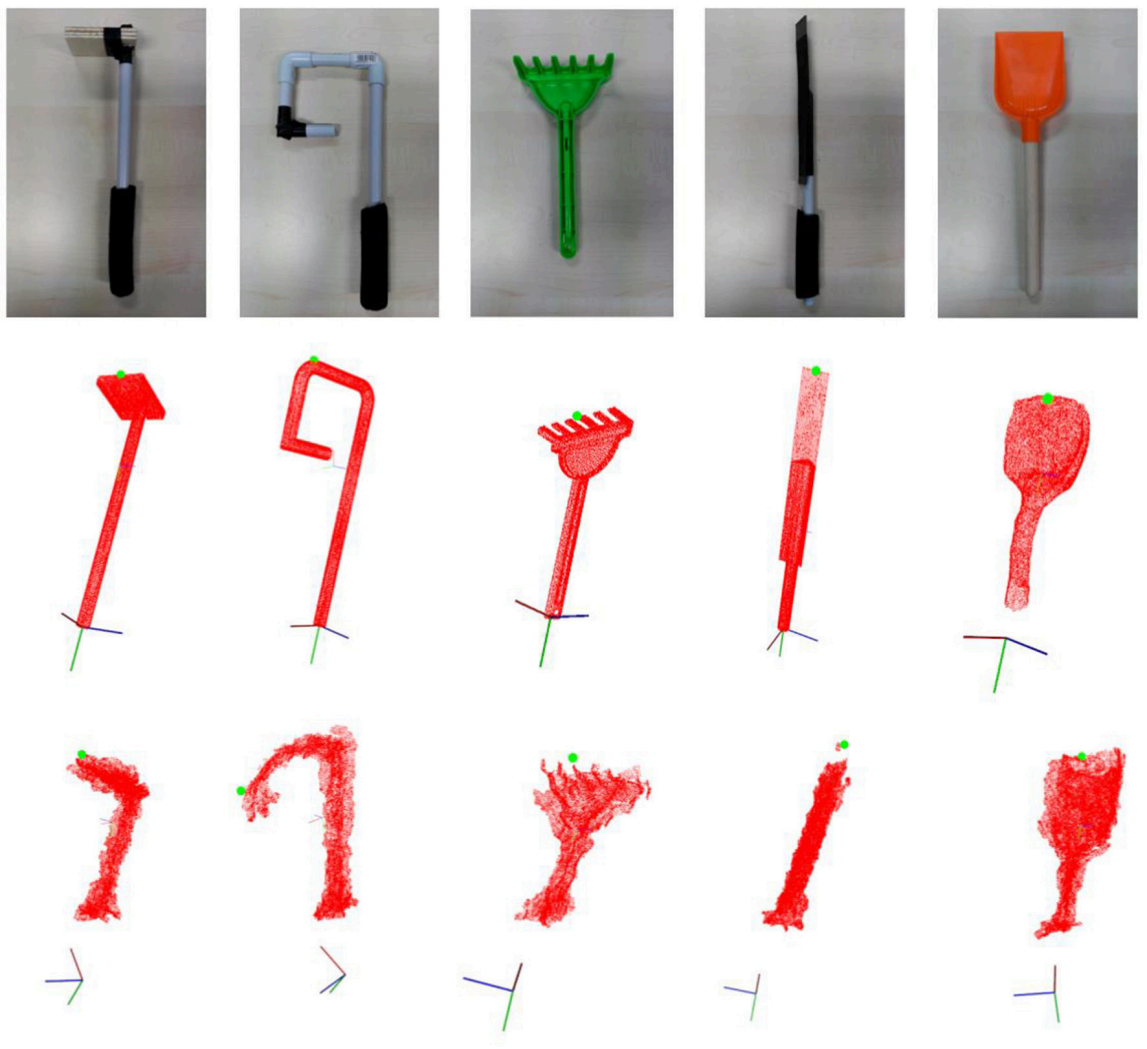
Results of the tooltip estimation process described in the text shown for a few example tools (top row), whose pointcloud has been achieved from from CAD models (middle row), or autonomously reconstructed (bottom row).

The estimated tool reference frame **f** and tooltip for a small sample of tools can be observed in Figure 2, where it can be observed that the estimated tooltip coincides to what most people would consider to be the tooltip of those tools.

In our code, this definition is implemented by the function findTooltipSym, which computes the tooltip location based on the information from the tool planes provided by the previous steps.

## 5. Tool pose estimation

The methods described in sections 3 and 4 allow the robot to reconstruct a tool's geometry and estimate its pose even in the case of previously unseen tools. However, this is a time consuming approach that is not necessary if a 3D pointcloud model of the tool or tool category is already available, either from a CAD model or from a previous reconstruction. For these cases, in this section we introduce a fast and reliable method for pose estimation, based on the alignment of the available model with a single partial view reconstruction to the tool in the robot's hand, implemented in the function findPoseAlign.

Qualitatively, the *tool pose* represents the way in which the tool is being grasped with respect to the hand's reference frame. Numerically, we can express the tool pose in terms of the 4 × 4 roto-translation **Pose Matrix** *P* required to transform the hand reference frame < *H* > frame to any reference frame intrinsic to the tool < *T* >, that is,

(10) $$\begin{array}{r} <T>\;=P<H> \end{array}$$

The hand reference frame < *H* > is defined by the robot kinematics. The tool reference frame < *T* > applied can be arbitrarily chosen, as long as it is coherent among all the tools that can be considered, as the Pose is expressed in relative terms.

This means that *P* can also be understood as the required transformation to align a tool 3D model from its canonical pose to the pose in which is the tool is being held by the robot, given by the oriented pointcloud model. In this work, this transformation is estimated by aligning the available model of the tool with a partial reconstruction obtained through iCub's disparity.

**Figure 3:**
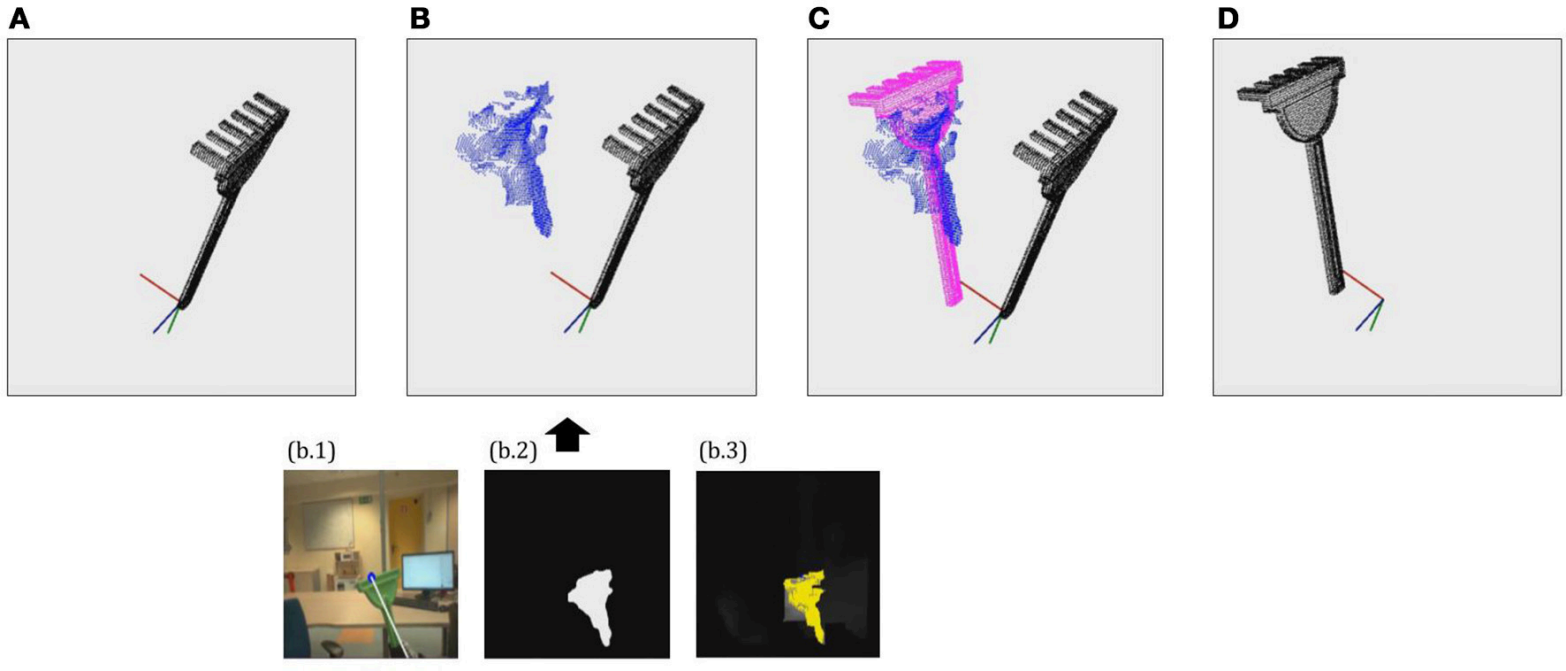
Example of the tool pose estimation through alignment process. **(A)** Load 3D pointcloud model on canonical pose. **(B)** Extract partial reconstruction using seg2cloud model (segmentation + depth estimation). **(C)** Find Pose Matrix *P* by aligning 3D model to partial reconstruction. **(D)** Obtain oriented pointcloud model by applying *P* to the 3D model.

To that end, iCub first applies the method described in section 2 to identify the tool instance or category and load the corresponding model. Then, it fixates the gaze on the tool's effector and extracts a partial pointcloud reconstruction, using the same methods applied on each of the exploration poses considered for tool reconstruction, as detailed in section 3. Then, the ICP algorithm is applied in order to align the pointcloud model loaded from memory to the partial reconstruction just obtained. Finally, the alignment matrix returned by the ICP is checked to assess whether it corresponds to a feasible grasp pose in terms of translation from the origin and rotation in Z and X axes. If the alignment estimated by ICP corresponds to a feasible grasp, then the returned alignment matrix is assigned to *P*, and applied to transform the canonical pointcloud model available in memory in order to obtain the oriented pointcloud model. This process can be observed in Figure 3.

Thereby, after the pose estimation process iCub has explicit information about the precise geometry and pose of the tool in its hand. Therefore, it can apply the method described in section 4.3 to determine the position of the tooltip with respect to the robot's hand reference frame, and hence extend the kinematics of the robot to incorporate the tip of the tool as the new end-effector for further action execution.

## 6. Conclusion

In the present paper we have introduced the concept of tool incorporation, that is, the process whereby the iCub robot is able to recognize a tool, estimate its geometry, pose and tooltip, and use this information to use the tool as its new end-effector. In particular, we have introduced a repository which implements a set of interconnected methods to perform such tasks in a fast and reliable way on iCub platform. By applying these methods, the robustness of the desired tool use behaviors as well as the ease of implementation can be substantially increased, by reducing the necessity of applying predefined parameters to represent the tools.

Despite its clear advantages, this approach does however suffer from a few limitations. On the one hand, it only works properly with radial tools where handle and effector are clearly distinct and are grasped radially (in the direction of the iCub's thumb). On the other, the 3D reconstruction quality, while generally enough to estimate the tool frame and the tooltip, does yield relatively noisy models. These issues clearly demand further work on tool incorporation mechanisms in order to facilitate robotic tool use.

## Author contributions

TM is the main author of the code and paper. VT provided technical guidance and assistance, and reviewed both the code and the paper. LN provided high-level supervision, and reviewed the paper before submission.

### Conflict of interest statement

The authors declare that the research was conducted in the absence of any commercial or financial relationships that could be construed as a potential conflict of interest. The reviewer JAG and handling Editor declared their shared affiliation.

## Footnotes

^1^https://github.com/robotology/segmentation/tree/master/dispBlobber

^2^We refer as radial tools to tools consisting of clearly distinct handle and effector, which are grasped from the handle with the thumb toward the effector of the tool (called radial grip).
